# Sex differences in quality of life of patients following percutaneous coronary intervention in Vietnam

**DOI:** 10.1007/s11136-022-03237-5

**Published:** 2022-08-29

**Authors:** Hoa Vu, Richard Norman, Ngoc M. Pham, Hoai T. T. Nguyen, Hung M. Pham, Quang N. Nguyen, Loi D. Do, Christopher M. Reid

**Affiliations:** 1 Thai Nguyen University of Medicine and Pharmacy, Thai Nguyen, Vietnam; 2 School of Public Health, Curtin University, Perth, WA Australia; 3 Menzies Institute for Medical Research, University of Tasmania, Hobart, Australia; 4 Vietnam National Heart Institute, Hanoi, Vietnam

**Keywords:** Percutaneous coronary intervention, Sex differences, Quality of life, Vietnam

## Abstract

**Purpose:**

Data regarding the health-related quality of life (HRQoL) in men and women after percutaneous coronary intervention (PCI) in low-and-middle incomes countries remains scarce. To investigate sex difference in the HRQoL at 30 days and 12 months post PCI in Vietnam.

**Methods:**

We used data from a single percutaneous coronary registry established in the Vietnam National Heart Institute. The HRQoL was measured using the EQ-5D-3L instrument via telephone interviews, and information about demographics, clinical presentation and procedures was obtained through face-to-face interviews and medical records. The HRQoL between men and women were compared using independent samples *t* tests, the Mann–Whitney *U* test or univariate/multivariate logistic regression.

**Results:**

There were 866 patients included in this cohort, with the proportion of women being nearly half of men (32.1 vs 67.9%). Women were 3.5 years older, had lower income and educational levels but greater prevalence of cardiovascular risk factors. At 30 days, women significantly had more problems in mobility, personal care and pain/discomfort compared to men. At 12 months, women had more problem than men in usual activity. The geometric means of HRQoL was lower in women at 30 days, but not in 12 months. From 30 days to 12 months after discharge, women showed better recovery in mobility compared to men, but not in other dimensions.

**Conclusions:**

In this first insight of sex differences in HRQoL following PCI in Vietnam, women appeared to have worse quality of life, especially at 30 days after discharge but they showed better recovery in mobility at 12 months compared to men.

**Supplementary Information:**

The online version contains supplementary material available at 10.1007/s11136-022-03237-5.

## Introduction

Studies regarding the effectiveness of medical treatments and patient recovery from cardiac interventions have attracted growing interest. While mortality and objective measures of morbidity are traditionally considered key clinical outcome measures, health-related quality of life (HRQoL) has been increasingly utilized in contemporary research due to its capability to provide patient-reported outcomes covering both symptoms and the functional impact of the illness and its treatments [1–3]. Patients therefore provide their own perspectives in defining health status, both in terms of how they feel (distress and well-being) and how they evaluate their own health and future prospects [4, 5].

Sex differences in clinical outcomes after percutaneous coronary intervention (PCI) have been widely documented in current literature, and typically suggest that women have worse post procedural outcomes than men (e.g., higher mortality and morbidity rates) [6–11]. Among studies that have addressed the gender differences in HRQoL outcomes after PCI, the majority of them have observed poorer post-procedure HRQoL in women patients, especially in the short and medium-term [7, 8, 12–14], while some publications reported no significant differences between the sexes in HRQoL in the long-term post procedure (3 and 10 years) [6, 12]. Explanations for the sex differences in outcomes in these studies include biological factors such as age and cardiovascular risk factors, and social factors such as social support and health care access [7, 15]. However, such findings are inevitably context-specific, meaning it remains unclear that such explanations can be applied into different populations and cultures. Hence, more comprehensive studies are required to explore this aspect, especially in limited resources countries where the health of women is often prioritised less than that of men.

Our study is the first to utilize data from a PCI registry in Vietnam to examine the HRQoL between men and women at 30 days and 12 months post discharge.

## Method

### Study setting

This study uses data from a PCI registry conducted from September 2017 to May 2018 in Vietnam National Heart Institute (VNHI), Hanoi, Vietnam. Details of the PCI registry have been previously described [16–19]. Briefly, the pilot hospital-based registry adapted the data collection forms from a long-standing registry in Australia to collect information on demographic, clinical and procedural information, and outcomes of patients who underwent PCI at VNHI [20, 21]. After discharge from the index PCI, patients were followed up at 30 days and 12 months for information about their health status and quality of life. Data were collected through interviewing patients, extracting medical records and reading secured disks at baseline. At follow-up visits, data collection was conducted via face-to-face and supplemented with telephone interviews. Ethics approval was obtained from the Curtin Human Research Ethics Committee before the commencement of data collection (HRE 2017-0378). Patients were aware of the right to opt out from the study without any treatment impact. A team of specifically trained local investigators at VNHI was responsible for data collection.

The study captured 1022 patients from the PCI registry. For the purpose of HRQoL analysis, all patients who had data available and were alive 12 months after the index PCI were included in this study. Those with missing HRQoL data at 30 days (*n* = 43) and 12 months (*n* = 53) and those had died in the 1 year subsequent to hospital discharge (*n* = 60) were excluded. Essential characteristics between participants who were lost to follow-up (*n* = 96) and those who were not (*n* = 866) were compared and found that the characteristics between two groups are similar with the exception of age. Therefore, eliminating 96 patients lost to follow-up was unlikely to significantly affect the results reported in the paper. A final sample of 866 patients remained for the present analysis.

### Quality of life at 30 days and 12 months

The primary endpoint was patients’ HRQoL at 30 days and 12 months according to sex. HRQoL was assessed using the EQ-5D-3L, an instrument for health status measurement developed by the Euro HRQoL group [22]. The EQ-5D-3L is composed of the following dimensions: mobility; personal care; usual activities; pain/discomfort; and anxiety/depression. Each dimension has three response levels: no, some or extreme problems (e.g., unable to walk, extreme pain/discomfort). There are 243 theoretically possible health status of patients in the EQ-5D-3L, in which each health status is referred to as a health state. Each patient response to the EQ-5D-3L was converted to an index score, using the Malaysian value set. Vietnam does not have a value set for the instrument, and we selected the Malaysian data due to the geographic, socioeconomic and cultural similarity between the Malaysian and Vietnamese populations [23]. The higher EQ-5D-3L score indicates the higher health status for patients. Additionally, patients were asked to provide a self-rating health status based on a scale of 0–100, the EQ-Visual Analogue Scale (VAS), in which a higher score indicated better self-assessed health. The change in HRQoL of patients from 30 days to 12 months were calculated based on their answers of HRQoL at two follow-ups and categorised into “no change”, “get worse”, and “get better”. Get better was defined if a patient reported better health status at 12 months in comparison with 30 days (extreme/some problems to no problems; extreme to some problems). Get worse was defined if a patient claimed worse health status at 12 months compared to that at 30 days (no problem to some or extreme problems; some problems to extreme problems).

### Clinical presentations

ST-elevation myocardial infarction (STEMI) was defined as the elevation of cardiac biomarkers and new or presumed new ST-segment elevation in two or more contiguous ECG leads. Non-ST-elevation myocardial infarction (NSTEMI) was diagnosed in the presence of elevated cardiac biomarkers and at least one of either, changes in ECG (ST-segment depression or T wave abnormalities) or ischemic symptoms [24]. Unstable angina (UA) was identified in the presence of prolonged chest pain without cardiac enzyme elevation [25].

The strategy for the specific coronary intervention (e.g., choice of entry location, stent, medication) was at the discretion of the interventionists. Injured lesion segments were identified and coded following the definition of the coronary tree segments developed in the SYNTAX study (26) and guidelines for the lesion type of the American College of Cardiology/American Heart Association (ACC/AHA) [27]. A procedure was considered successful if there was a residual stenosis of less than 10% following coronary stenting and the rate of coronary blood perfusion of Thrombosis in Myocardial Infarction (TIMI) 2 or 3 flow. Bleeding was classified by the Bleeding Academic Research Consortium (BARC) criteria [28], while major bleeding was defined by any transfusion or by a drop-in haemoglobin ≥ 3.0 g/dl.

### Statistical analysis

Categorical data on baseline and procedural characteristics between men and women were presented as numbers and percentages and analysed using Fisher’s exact or Chi-square tests as appropriate. Mean ± SD and independent samples *t* tests were used to present and analyse the normally distributed continuous data, while skewed variables were presented as medians and analysed using the Mann–Whitney *U* test or median test. Answers in each HRQoL dimension were then regrouped into two categories only: no problem and presence of a problem with “some” and “extreme” answers combined. Univariate and multivariate logistic regression were performed to obtain crude and adjusted odds ratios and 95% confidence intervals for sex in each HRQoL dimension. Age, low-income, lower education, STEMI, hypertension, diabetes mellitus, hyperlipidemia, previous CABG, previous PCI, left main disease have been utilized for adjusting the differences in men and women. The HRQoL index scores were transformed due to highly skewed data and then back transformed to obtain the geometric means. To evaluate the effect size for the differences in VAS, Cohen’s D has been applied for crude model and epsilon has been utilized for the adjusted model. To assess the change in HRQoL from 30 days to 12 months between men and women at three states (get worse, no change and get better), a Mann Whitney *U* test was undertaken. All *p* values were two-tailed with statistical significance defined as *p* ≤ 0.05. All statistical analyses were conducted using SPSS Version 27.0 for Windows.

## Results

### Baseline and procedural characteristics

Characteristics of male and female patients are shown in Table 1. Of 866 patients, the proportion of women was less than half of men (32.1 vs. 67.9%). Compared with men, women were 3.5 years older, had a lower income and educational level, had a greater prevalence of previous cardiovascular risk factors but reported a lower prevalence of prior coronary artery revascularization as well as lesion in left main coronary artery (all *p* < 0.05).

**Table 1:** Baseline and procedural characteristics according to sex

	Total (*n* = 866)	Women (*n* = 278)	Men (*n* = 588)	P*
Age (years), mean ± SD	67.60 ± 9.82	70.00 ± 9.03	66.46 ± 9.98	< 0.001
Low-income^a^	648 (73.5)	235 (84.5)	413 (70.2)	< 0.001
Lower education^b^	521 (59.1)	181 (65.1)	340 (57.8)	0.041
BMI (kg/m^2^), mean ± SD	22.24 ± 2.93	21.96 ± 3.09	22.37 ± 2.85	0.056
Presentation				
STEMI	114 (12.9)	27 (9.7)	87 (14.8)	0.039
NSTEMI	136 (15.4)	47 (16.9)	89 (15.1)	0.504
Unstable angina	214 (24.3)	74 (26.6)	140 (23.8)	0.371
ACS	464 (52.6)	148 (53.2)	316 (53.7)	0.890
Stable CAD	402 (45.6)	130 (46.8)	272 (46.3)	
Medical history				
Hypertension	578 (65.5)	208 (74.8)	370 (62.9)	0.001
Diabetes mellitus	238 (27.0)	94 (33.8)	144 (24.5)	0.004
Hyperlipidaemia	270 (30.6)	106 (38.1)	164 (27.9)	0.002
Prior cerebral vascular disease	121 (13.7)	38 (13.7)	83 (14.1)	0.860
Current smoking	108 (12.2)	1 (33.3)	107 (25.6)	0.760
Previous CABG	11 (1.2)	0 (0.0)	11 (1.9)	0.022
Previous PCI	309 (35.0)	80 (28.8)	229 (38.9)	0.004
Tests prior to PCI				
Left ventricular ejection fraction ≤ 40%	86 (9.8)	24 (9.4)	62 (12.0)	0.292
Moderate to severe renal impairment a	15 (1.7)	3 (1.1)	12 (2.1)	0.311
Procedural characteristics				
Radial access	685 (77.7)	220 (79.1)	465 (79.1)	0.985
Left main disease	93 (10.5)	17 (6.1)	76 (12.9)	0.003
Lesion type B2 and C	822 (93.2)	263 (94.6)	559 (95.1)	0.772
≥ 2 Stents per lesion	359 (40.7)	105 (37.8)	254 (43.2)	0.130
PCI with ≥ 2 lesions	182 (20.6)	57 (20.5)	125 (21.3)	0.799
Stent used	856 (97.1)	276 (99.3)	580 (98.6)	0.410
Balloon used only	3 (0.3)	1 (0.4)	2 (0.3)	0.963
Procedural success	860 (97.5)	277 (99.6)	583 (99.1)	0.416
In hospital major bleeding	15 (1.7)	8 (2.9)	7 (1.2)	0.076

*BMI* body mass index, *STEMI* ST-elevation myocardial infraction, *NSTEMI* non-ST-elevation myocardial infraction, *CABG* coronary artery bypass grafts, *PCI* percutaneous coronary intervention

^*^Based on the *χ*^2^ test

^a^Individual monthly income < 216 USD with the exchange rate of 23.150 VND

^b^Education from primary to high school

### 30 days and 12 months quality of life post PCI

HRQoL of men and women are presented in Tables 2 and 3. At 30 days, over 80% of patients reported having no problem in mobility, personal care, and anxiety/depression, and more than 75% of them reported experiencing some problems in usual activity and moderate pain/discomfort (Table 2). Similarly, the proportions of patients without problems in mobility, personal care, usual activity and anxiety/depression were high (> 80%) at 12 months. Approximately two third of patients reported they had no pain/discomfort and one third reporting moderate pain/discomfort (Table 3). The geometric means of HRQoL index score at 30 days and 12 months are 0.780 (95% confidence interval: 0.770–0.790) and 0.866 (0.857–0.874), respectively. The means of VAS at 30 days and 12 months are 69.69 (68.08–69.31) and 72.49 (71.66–73.33), respectively. HRQoLAt 30 days, women were significantly more likely than men to report a problem in 3 dimensions of EQ-5D-3L. Specifically, women reported higher mobility problems relative to men (21.6% vs 11.6%), higher personal care problems (12.6% vs 7.0%,) and higher pain or discomfort compared to men (81.7% vs 79.8%). Women had significantly poorer mobility (adjusted OR 1.818, 95% CI 1.218–2.713, *p* = 0.003), higher personal care problems (adjusted OR 1.590, 95% CI 0.987–2.563, *p* = 0.057), and higher pain or discomfort experience (OR 1.574, 95% CI 1.022–2.425, *p* = 0.04) compared with men. In addition, the adjusted geometric means of HRQoL index score was lower in women (0.763 vs 0.788, *p* = 0.031), despite similar adjusted self-rate EQ-VAS score between men and women at 30 days.

**Table 2:** 30-day quality of life (HRQoL) post PCI according to sex

Variable	Total, *n* (%)	Women, *n* (%)	Men, *n* (%)	Crude		Adjusted^b^	
				OR (95% CI)a	*P* value*	OR (95% CI)	*P* value*
HRQoL mobility							
No problem	731 (84.4)	216 (77.7)	515 (87.6)	1.00 (reference)		1.00 (reference)	
Some problem	128 (14.8)	60 (21.6)	68 (11.6)	2.025 (1.393–2.943)	< .001	1.818 (1.218–2.713)	0.003
Unable to walk around	7 (0.8)	2 (0.7)	5 (0.9)				
HRQoL personal care							
No problem	778 (89.9)	238 (85.6)	540 (91.8)	1.00 (reference)		1.00 (reference)	
Some problem	76 (8.8)	35 (12.6)	41 (7.0)	1.891 (1.210–2.955)	0.005	1.590 (0.987–2.563)	0.057
Unable to wash/dress self	12 (1.4)	5 (1.8)	7 (1.2)				
HRQoL usual activity							
No problem	179 (20.7)	48 (17.3)	131 (22.3)	1.00 (reference)		1.00 (reference)	
Some problem	667 (77.0)	221 (79.5)	446 (75.9)	1.374 (0.952–1.982)	0.09	1.193 (0.807–1.764)	0.377
Unable to perform usual activities	20 (2.3)	9 (3.2)	11 (1.9)				
HRQoL pain/discomfort							
No pain/discomfort	128 (14.8)	31 (11.2)	97 (16.5)	1.00 (reference)		1.00 (reference)	
Mod pain/discomfort	696 (80.4)	227 (81.7)	469 (79.8)	1.574 (1.022–2.425)	0.04	1.360 (0.860–2.153)	0.189
Extreme pain/discomfort	42 (4.8)	20 (7.2)	22 (3.7)				
HRQoL anxiety/depression							
No anxiety/depression	729 (84.2)	228 (82.0)	501 (85.2)	1.00 (reference)		1.00 (reference)	
Mod anxiety/depression	132 (15.2)	50 (18.0)	82 (13.9)	1.263 (0.862–1.849)	0.23	1.180 (0.786–1.772)	0.424
Extreme anxiety/depression	5 (0.6)	0 (0.0)	5 (0.9)				
HRQoL EQ-5D Index Score, geometric means (95% CI)							
Crude	0.780 (0.770–0.790)	0.755 (0.738–0.773)	0.792 (0.779–0.805)		0.001		
Adjusted^b^	NA	0.763 (0.744–0.781)	0.788 (0.775–0.801)				0.031
HRQoL own health state today (VAS), means (95% CI)							
Crude	69.69 (68.08–69.31)	68.26 (67.24–69.27)	69.77 (69.07–70.47)		0.016		
Adjusted^b^	NA	68.64 (67.60–69.67)	69.59 (68.89–70.28)				0.144
Effect size				Cohen’s *d*: 0.175		*ε*^2^: 0.001	

*I* confidence interval, *OR* odds ratio, *NA* not applicable, *VAS* visual analogue scale

^*^P for difference in point estimates between women and men based on multiple regression analysis

^a^The main exposure was sex in which men were taken as the referent group; the outcome was each dimension of HRQoL, with a problem being defined as the combination of “some” and “extreme” category

^b^Adjusted for age, low-income, lower education, STEMI, hypertension, diabetes mellitus, hyperlipidemia, previous CABG, previous PCI, left main disease

**Table 3:** 12 months quality of life (HRQoL) post PCI according to sex

Variable	Total, *n* (%)	Women, *n* (%)	Men, *n* (%)	Crude		Adjusted^b^	
				OR (95% CI)^a^	*P* value*	OR (95% CI)	*P* value*
HRQoL mobility							
No problem	731 (84.4)	232 (83.5)	499 (84.9)	1.112 (0.754–1.639)	0.593	0.95 (0.63–1.44)	0.820
Some problem	132 (15.2)	45 (16.2)	87 (14.8)				
Unable to walk around	3 (0.3)	1 (0.4)	2 (0.3)				
HRQoL personal care							
No problem	840 (97.0)	266 (95.7)	574 (97.6)	1.850 (0.844–4.054)	0.124	1.48 (0.63–3.47)	0.374
Some problem	23 (2.7)	11 (4.0)	12 (2.0)				
Unable to wash/dress self	3 (0.3)	1 (0.4)	2 (0.3)				
HRQoL usual activity							
No problem	705 (81.4)	217 (78.1)	488 (83.0)	1.372 (0.961–1.959)	0.082	1.48 (1.01–2.16)	0.046
Some problem	157 (18.1)	60 (21.6)	97 (16.5)				
Unable to perform usual activities	4 (0.5)	1 (0.4)	3 (0.5)				
HRQoL pain/discomfort							
No pain/discomfort	570 (65.8)	172 (61.9)	398 (67.7)	1.219 (0.959–1.738)	0.092	1.30 (0.94–1.78)	0.109
Mod pain/discomfort	282 (32.6)	100 (36.0)	182 (31.0)				
Extreme pain/discomfort	14 (1.6)	6 (2.2)	8 (1.4)				
HRQoL anxiety/depression							
No anxiety/depression	833 (96.2)	266 (95.7)	567 (96.4)	1.218 (0.590–2.513)	0.593	1.29 (0.60–2.78)	0.513
Mod anxiety/depression	33 (3.8)	12 (4.3)	21 (3.6)				
Extreme anxiety/depression	0 (0.0)	0 (0.0)	0 (0.0)				
HRQoL EQ-5D Index Score, geometric means (95% CI)							
Crude	0.866 (0.857–0.874)	0.856 (0.841–0.871)	0.870 (0.860–0.881)		0.12		
Adjusted^b^	NA	0.859 (0.844–0.874)	0.869 (0.859–0.879)				0.316
HRQoL own health state today (VAS), means (95% CI)							
Crude	72.49 (71.66–73.33)	70.77 (69.30–72.23)	73.31 (72.30–74.32)		0.005		
Adjusted^b^	NA	71.34 (69.83–72.85)	73.04 (72.02–74.06)				0.074
Effect size				Cohen’s *d*: 0.204		*ε*^2^: 0.003	

*OR* odds ratio, *CI* confidence interval, *VAS* visual analogue scale

^*^P for difference in point estimates between women and men based on multiple regression analysis

^a^The main exposure was sex in which men were taken as the referent group; the outcome was each dimension of HRQoL, with a problem being defined as the combination of “some” and “extreme” category

^b^Adjusted for age, low-income, lower education, STEMI, hypertension, diabetes mellitus, hyperlipidemia, previous CABG, previous PCI, left main disease

At 12 months, women were also more likely than men to report a problem in all 5 dimensions of EQ-5D-3L. The differences were found significantly in usual activity when women reported more problem with daily activities than men (adjusted OR 1.48, 95% CI 1.01–2.16, *p* = 0.046). There were no differences found in the adjusted geometric mean of the HRQoL index score and VAS of men and women at 12 months.

### Change in quality of life from 30 days to 12 months

**Fig. 1:**
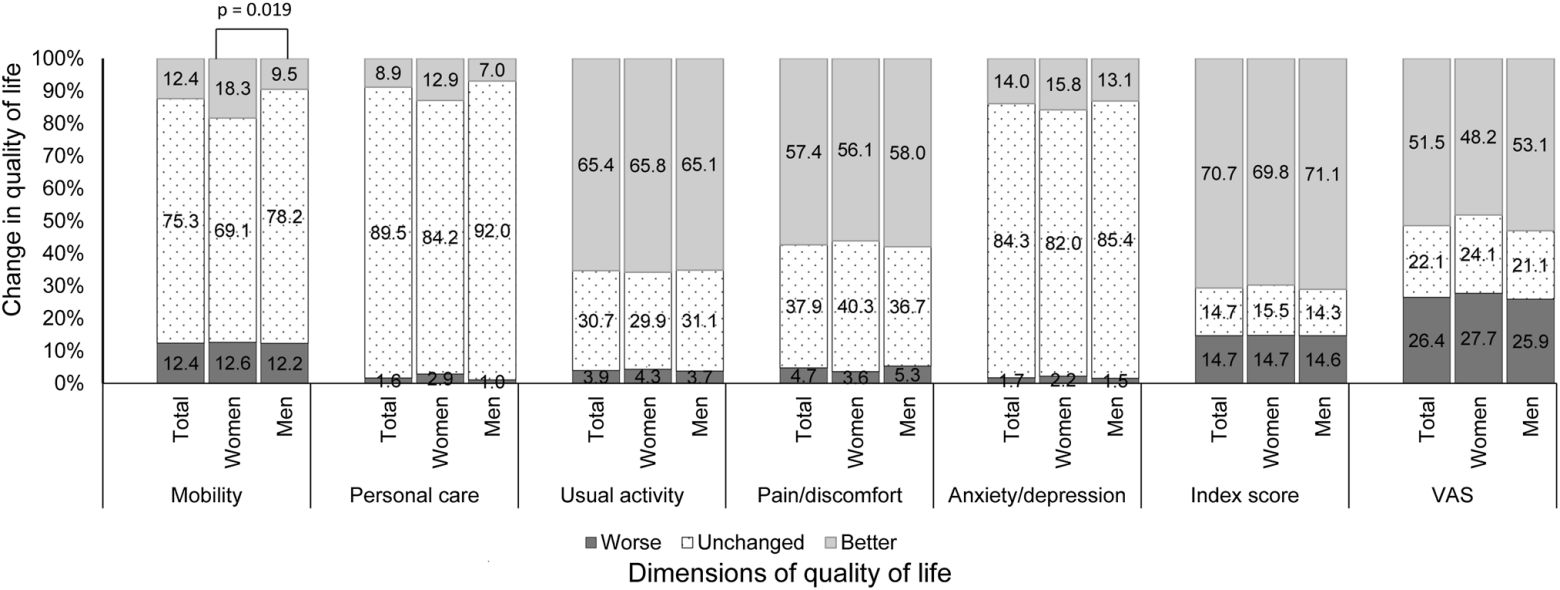
Change in quality of life from 30 days to 12 months. The respective number of subjects whose quality of life got worse, remained unchanged or got better in the total sample, women and men according to each dimension of quality of life was as follows: mobility (107, 652, 107; 35, 192, 51; and 72, 460, 65), personal care (14, 775, 77; 8, 234, 36; and 6, 541, 41), usual activity (34, 266, 566; 12, 83, 183; and 22, 183, 383), pain/discomfort (41, 328, 497; 10, 112, 156; and 31, 216, 341), anxiety/depression (15, 730, 121; 6, 228, 44; and 9, 502, 77), HRQoL index score (127, 127, 612; 41, 43, 194; and 86, 84, 418), visual analogue scale (VAS: 229, 191, 446; 77, 67, 134; and 152, 124, 132)

Figure 1 presents the changes in the dimensions of the EQ-5D, the HRQoL index score and the EQ-Visual Analogue Scale (VAS). Over 75% patients reported their health status being unchanged from 30 days to 12 months with respect to mobility, personal care, and anxiety/depression. In performing usual activity and experience of pain/discomfort, approximately two third of patients reported better outcomes from 30 days to 12 months. The change in HRQoL index score and self-rate VAS indicated that the majority of patients reported better outcome at 12 months.

Compared with men, women were significantly more likely to recover better in mobility (18.3% vs 9.5%, *p* = 0.019), with a suggestion of better recovery for personal care (12.9% vs 7.0%, *p* = 0.061). There were no significant differences between men and women regarding the change in other dimensions (i.e., usual activity, pain/discomfort and anxiety/depression), or HRQoL index score and VAS (*p* > 0.05).

## Discussion

This study is the first using PCI registry data at 30 days and 12 months to compare HRQoL after PCI between men and women in a low-and-middle income country where men are culturally more prioritized than women. The results indicated that women patients experienced poorer HRQoL after PCI in comparison with men. The differences were seen more clearly at 30 days than at 12 months post discharge, perhaps suggesting a higher recovery rate in the women patients. These findings contribute to the literature from developing countries in regard to HRQoL between men and women after an effective revascularization such as PCI. Further, they are potentially beneficial for defining an appropriate intervention strategy targeting patient outcomes after PCI in Vietnam. It appears that women are in greater need, particularly at 30 days suggesting that sex-specific health care approaches should be taken into consideration in providing care for PCI patients.

Available studies in literature that examined the sex differences in HRQoL outcome among PCI patients have utilized a range of HRQoL measurements. Using the same EQ-5D-3L instrument as our study, the Australian state-wide study including data from 30 cardiac centres reported poorer HRQoL outcome of women in comparison with men and confirmed being female was a significant predictor for poor HRQoL at 30 days [7]. In other studies using different HRQoL instruments such as Short-Form 12 questionnaire, 36-Items Short-Form Health Survey and the MacNew Heart Disease Health-Related QoL questionnaire, some reported general worse health condition in women [6, 12], while there were others indicating women were lower in specific areas of physical, disease-specific HRQoL and mental health [13, 29]. Findings in our study are consistent with previous findings regardless the HRQoL instruments, indicating that women had more problems in mobility, personal care and pain/discomfort at 30 days after PCI. In other studies that investigated the HRQoL outcomes between men and women after myocardial infarction or coronary artery diseases, similar results were documented. Women patients were more HRQoL disadvantaged than male counterparts at short and mid-terms such as 30 days, 1 year and 2.5 years [30–32]. The available evidence suggested that HRQoL declines with the presence and intensity of cardiovascular risk factors such as hypertension, lipid metabolism disorders, diabetes, and sedentary lifestyle. It was also indicated that the more cardiovascular factors present at the same time, the worse the HRQoL score [6, 33]. Furthermore, older age was considered as an associated factor with low HRQoL scores due to the restrictions and difficulties in familial, social and professional life for the elderly [6]. Our results were consistent with these explanations, showing that women undergoing PCI were older, lower in education and income, and had significantly higher rates of cardiovascular risks in comparison with men counterparts. However, our study did not indicate the associated factors for worse HRQoL outcomes, more dedicated studies need to address this issue in future and more sex-specific health care programs should be implemented.

Studies regarding HRQoL of PCI populations have also documented no sex differences in long-term follow-up and have shown that women improved better than men in HRQoL over time. It was reported that HRQoL differences between men and women PCI patients were seen in short and mid-terms such as 1 months and 6 months but not for longer terms such as 3 years or 10 years [6, 12]. Similarly, a study of Włodarczyk showed men performed higher in the physical and emotional aspects of life at 2 and 3 months follow-ups after myocardial infarction, but not at 12 months assessment [34]. This finding was reinforced in our study with no statistically significant difference in HRQoL of the men and women at 12 months. Regarding the HRQoL improvement of patients post discharge, findings remain controversial in current literature. While both sexs reported significant improvement over time, women were more likely to have better recovery in HRQoL [6]. Another study concluded that women patients were reported with slower physical improvement compared to men [29]. In our study, women were found to have better recovery in mobility compared to men. The reason for this change remains unclear and further research should be conducted to investigate this issue further.

This study has some limitations. Our results may not be generalisable to the entire Vietnamese adults because this study was based on registry data collected from only one leading cardiac hospital in Vietnam. Given the hospital is a relatively advanced provider of cardiac intervention, it may be that the outcomes observed elsewhere might be worse, and hence flagging an even greater need for better follow-up care in this patient group. Another limitation is that we could not collect HRQoL before PCI, therefore could not conclude the effect of PCI on HRQoL change. This was because many of the patients were admitted as emergency cases, and there was not an opportunity to collect data prior to PCI. In addition, due to the lack of specific value set for the EQ-5D-3L derived from preferences of the Vietnamese population, the Malaysian value set was used to generate the index score due to the similarity between the two populations. We strongly encourage the development of a specific HRQoL value set for the Vietnamese population to increase the precision of results obtained. Lastly, although potential confounding factors were adjusted, the effect of unmeasured confounders and/or residual confounding cannot be ruled out. This may have obscured the relation between gender and QoL measures, if any.

## Conclusion

Our study utilizing data from the first Vietnamese PCI registry provides an opportunity to investigate the sex differences in HRQoL post discharge. Overall, women obtained worse HRQoL after PCI in comparison with men. The differences were seen more clearly at 30 days than at 12 months post discharge, and women were reported to have better recovery in mobility compared to men.

## Supplementary Information

Below is the link to the electronic supplementary material.

Supplementary file1 (DOCX 38 kb)

## Abbreviations

ACS: Acute coronary syndrome
AMI: Acute myocardial infarction
CABG: Coronary artery bypass grafts
CHD: Coronary heart disease
HRQOL: Health related Quality of Life
NSTEMI: Non-ST-elevation myocardial infraction
PCI: Percutaneous coronary intervention
STEMI: ST-elevation myocardial infraction
UA: Unstable angina
VNHI: Vietnam National Heart Institute
