# Supplementary material for: Sex differences in quality of life of patients following percutaneous coronary intervention in Vietnam
Source: Qual Life Res. 2022 Aug 29;32(1):71–9. doi: 10.1007/s11136-022-03237-5 (PMC9829621; doi:10.1007/s11136-022-03237-5)
Supplement: Supplementary file 1 — Supplementary file1 (DOCX 38 kb) [file 11136_2022_3237_MOESM1_ESM.docx]

**Title: Gender differences in quality of life of patients following percutaneous coronary intervention in Vietnam**

**Table 1: Baseline and procedural characteristics of lost and non-lost participants**

|  | Non-lost (n=866) | Lost (n=96) | P* | Effect size** |
| --- | --- | --- | --- | --- |
| Age (years), mean ±SD | 67.9 ± 10.1 | 71.8 ±10.8 | <0.001; | Cohen’s d =0.37 |
| Low-income ^a^ | 22 (2.6) | 2 (2.1) | 0.76 | 0.797 (0.184-3.442) |
| Lower education ^b^ | 339 (40.1) | 45 (46.9) | 0.20 | 0.758 (0.496-1.158) |
| BMI (kg/m2), mean ±SD | 22.2 ± 3.0 | 22.2 ± 3.0 | 0.93 | Cohen’s d=0 |
| Presentation |  |  |  |  |
| STEMI | 135 (16.0) | 13 (13.5) | 0.54 | 0.848 (0.460-1.565) |
| NSTEMI | 148 (17.5) | 18 (18.8) | 0.76 | 1.120 (0.651-1.925) |
| Unstable angina | 193 (22.8) | 21 (21.9) | 0.84 | 0.914 (0.550-1.521) |
| Medical history |  |  |  |  |
| Hypertension | 563 (66.5) | 64 (66.7) | 0.98 | 1.002 (0.641-1.567) |
| Diabetes mellitus | 228 (27.0) | 22 (22.9) | 0.40 | 0.798 (0.485-1.315) |
| Hyperlipidaemia | 248 (29.3) | 27 (28.1) | 0.81 | 0.927 (0.581-1.481) |
| Prior cerebral vascular disease | 124 (14.7) | 11 (11.5) | 0.40 | 0.760 (0.394-1.464) |
| Previous CABG | 10 (1.2) | 1 (1.0) | 0.90 | 0.901 (0.114-7.116) |
| Previous PCI | 285 (33.7) | 38 (39.6) | 0.25 | 1.242 (0.806-1.914) |
| Tests prior to PCI |  |  |  |  |
| Left ventricular ejection fraction ≤ 40% | 681 (87.4) | 76 (86.4) | 0.78 | 0.901 (0.473-1.716) |
| Moderate to severe renal impairment | 19 (2.3) | 5 (5.2) | 0.083 | 2.435 (0.888-6.676) |
| Procedural characteristics |  |  |  |  |
| Radial access | 669 (79.1) | 82 (85.4) | 0.14 | 1.725 (0.957-3.107) |
| Left main disease | 54 (6.4) | 9 (9.4) | 0.27 | 1.158 (0.559-2.399) |
| Lesion type B2 and C | 800 (94.6) | 90 (93.8) | 0.74 | 0.841 (0.350-2.025) |
| ≥ 2 Stents per lesion | 351 (41.5) | 41 (42.7) | 0.82 | 1.033 (0.674-1.582) |
| PCI with ≥ 2 lesions | 163 (19.3) | 21 (21.9) | 0.79 | 1.164 (0.697-1.945) |
| Stent used | 835 (98.7) | 95 (99.0) | 0.83 | 0.818 (0.104-6.407) |
| Balloon used only | 3 (0.4) | 1 (1.0) | 0.33 | 3.028 (0.312-29.400) |
| Procedural success | 838 (99.1) | 96 (100.0) | 0.34 |  |
| In hospital major bleeding | 17 (2.0) | 3 (3.1) | 0.47 | 1.611 (0.463-5.600) |

BMI: Body Mass Index; STEMI: ST- elevation myocardial infraction; NSTEMI: Non-ST- elevation myocardial infraction; CABG: Coronary artery bypass grafts; PCI: Percutaneous coronary intervention

^a^ Individual monthly income < 216 USD with the exchange rate of 23.150 VND; ^b^ Education from primary to high school. * Based on the χ² test; ** Based on the Cohen’s d and OR and 95% CI of simple logistic regression with non-lost is the referent group.
